# s ·nr: a visual analytics framework for contextual analyses of private and public RNA-seq data

**DOI:** 10.1186/s12864-018-5396-0

**Published:** 2019-01-24

**Authors:** Paul Klemm, Peter Frommolt, Jan-Wilhelm Kornfeld

**Affiliations:** 1 Max Planck Institute for Metabolism Research, Gleuler Str. 50, Cologne, 50931 Germany; 2 Indivumed Group, Falkenried 88, Bldg. D, Hamburg, D-20251 Germany; 3 University of Southern Denmark, Department of Biochemistry and Molecular Biology, Campusvej 55, Odense, DK-5230 Denmark

**Keywords:** Next-generation sequencing, RNA-seq, Analysis workflow, Visual analytics, Visualization, GUI, GO analysis, Gene filtering, Differentially expressed genes

## Abstract

**Background:**

Next-Generation Sequencing (NGS) has been widely accepted as an essential tool in molecular biology. Reduced costs and automated analysis pipelines make the use of NGS data feasible even for small labs, yet the methods for interpreting the data are not sophisticated enough to account for the amount of information.

**Results:**

We propose s ·nr, a Visual Analytics tool that provides simple yet powerful visual interfaces for displaying and querying NGS data. It allows researchers to explore their own data in the context of experimental data deposited in public repositories, as well as to extract specific data sets with similar gene expression signatures. We tested s ·nr on 1543 RNA-Seq based mouse differential expression profiles derived from the public ArrayExpress platform. We provide the repository of processed data with this paper.

**Conclusion:**

s ·nr, easily deployable utilizing its containerized implementation, empowers researchers to analyze and relate their own RNA-Seq as well as to provide interactive and contextual crosstalk with data from public repositories. This allows users to deduce novel and unbiased hypotheses about the underlying molecular processes.

**Demo:**

Login demo/demo: snr.sf.mpg.de (Tested with Google Chrome)

**Electronic supplementary material:**

The online version of this article (10.1186/s12864-018-5396-0) contains supplementary material, which is available to authorized users.

## Background

Next-Generation Sequencing (NGS) has been established as a state-of-the-art tool in molecular biology. Whole transcriptome shotgun sequencing (RNA-Seq) is a popular tool used even by small labs to shed light on complex metabolic processes. Canonical algorithms aligning the raw shotgun reads to the genome and transcriptome of species are well established and operate fully automated [1]. The differential gene or transcript expression between experimental groups can also be derived using established algorithms [2]. For interpreting the analysis output, which often comes in the form of spreadsheets containing a large number of differentially expressed genes, researchers often fall back to basic office spreadsheet applications, such as Microsoft Excel or Apple Numbers. These tools lack custom-tailored data exploration features and are slow because they are not built for use-cases with such large data. This leads to inconsistent analysis procedures, unobserved patterns in the data (e.g. similarity of divergently expressed gene profiles across data sets), potentially wrong conclusions and a lot of frustration for biomedical researchers not trained in harnessing additional analytical workflows written in R, python or other languages. We propose s ·nr (pronounced “*sonar*”), a Visual Analytics tool empowering molecular biologists to explore their RNA-Seq experiments and shed light on patterns in the data. Our contributions are:

- A Visual Analytics tool based on feedback of domain experts to provide means of exploring vast NGS RNA-Seq datasets.
- Providing an exploration tool covering important aspects of interpreting the data without relying on third-party front-ends.
- Empowering researchers to interrogate a large number of RNA-Seq experiments to efficiently detect similar or diverging patterns.
- Providing means of identifying related NGS datasets in public repositories, despite being discordant in the interrogated model organism, tissue type or disease context.
- An open-source web-based implementation that can be easily set up using containerized deployment.

## Implementation

In this section we describe how we went from a thorough requirement analysis that defines the key functionality of s ·nr to the selection of web-based technologies and containerized solutions that allow for easy deployment and scalability of the tool.

### Requirement analysis

We conducted questionnaire-guided interviews to derive information about *task*, *context* and *user* with eight molecular biologists at all career stages working with RNA-Seq data. The questions ranged from research background, how RNA-Seq data is incorporated in that context and which tools support the data exploration. The protocol for the interview and the associated questionnaire is outlined in Additional file 1.

**Task:** From the interviews, we derived the following *tasks*:

- Gene Ontology (GO) [3] analysis: Finding relevant signaling pathways and disease ontologies that are enriched for the differentially expressed (DE) genes.
- Filter regulated genes: Select genes that are differentially regulated by setting interactive and cumulative thresholds.
- Compare datasets: Detect consistent regulation patterns across datasets.

As result of the interviews, we decided to focus on the *tasks* that were almost uniformly mentioned: *Performing GO analyses*, *filtering the genes* and *comparing datasets*.

**Context:** We concluded that many shortcomings in the analysis workflow arise from the *context* of the analysis (i.e. applying tools that are not suitable for this data). All interviewees use spreadsheet applications to process differential expression tables. Due to the size of the imported data, the software tends to be slow and unresponsive. Spreadsheet applications provide a direct interface to the expression tables, but also pose the risk of unintended modifications to the data. For comparing data sets, the problem is even more severe. Multiple datasets of the size of standard RNA-Seq results concatenated to large summary tables render such applications unresponsive. By an untrained molecular biologist, public repositories are usually queried based on meta-data only, e.g. same tissue from the same species and same condition as the own experiment. Functional similarity of the domain experts data with public data cannot be computed with the tools available. For incorporating GO term analysis or other functional analyses, the researchers have to use web-based interfaces by uploading their results to services typically hosted on servers in foreign countries with different data protection laws creating problems when handling sensitive data and research idea scooping.

**User:** As mentioned above, methods custom-tailored to NGS data, such as GO analyses, are often available either as web-interface or as Application Programming Interface (API) for programming languages such as R or Python. The web-interfaces typically expect a precise input format. The type conversion can prove difficult for researchers with limited coding experience, leading to time-consuming copy & paste sessions that are highly error-prone. Therefore we divided the domain experts into two different groups: those (1) with and (2) without programming skills to perform data wrangling and scripting for method APIs. To account for both groups, we have to simplify access to thirds-party tools. We have to account for data integrity and security by not sending information to third-party services.

Detailed results of the questionnaire as well as commonly used tools and plots can be found in Additional file 2.

### Analysis workflow design

The s ·nr workflow relies on Visual Analytics (VA), which combines data analytics techniques with interactive data visualization to derive insights into complex data sets [4]. The VA *mantra* defined by Keim et al. [5] is defined by four steps, **(1) analyze first** (rank information), **(2) show the important**, **(3) zoom, filter and analyze further** and show **(4) details on demand**. This workflow acts in iterative analysis workflow loops, allowing observations that trigger new hypotheses which in turn require more abstract views to go focus on other sections of the data. On top of the VA mantra, we established the following design principles for s ·nr: (1) Since the researchers have to interpret the vast data presented to them, we have to treat cognitive workload as a resource. This leads to (2) incorporating as *few* user interface elements and visual representations as possible. The power of the tool arises from (3) a high interactivity between few visual representations. We achieve this by incorporating brushing (selecting) and linking (broadcast selection) facilities [6] highlighting selected data entries in all data views.

**Data format.** We designed s ·nr to derive insight into differential gene expression using RNA-Seq data. This RNA-seq centric approach represents a mere proof-of-principle approach using a widely adopted class of NGS data. Any standard differential RNA-Seq dataset can be loaded into s ·nr. For further details, please refer to Additional files 3 and 4.

We incorporate the QuickNGS [7] platform (presented in this journal) for carrying out the alignment and differential expression calculation. QuickNGS allows us to easily process vast numbers of publicly available raw data automatically. By processing all data, private or public, with the same pipeline, we mitigate biases introduced by different read alignment and abundance estimation algorithms [8].

### Technology, optimizations and deployment

We built s ·nr using cutting-edge and scalable technologies. The system relies on a client-server structure allowing to shift computation-heavy operations to a powerful back-end and keep the requirements on the front-end machine low. Providing the interface as web-app allows for easy distribution, platform-independence and inherently is based on a client-server structure.

Our goal was to develop s ·nr as an easily deployable multi-user system that can be used production-ready in professional environments.

**Fig. 1:**
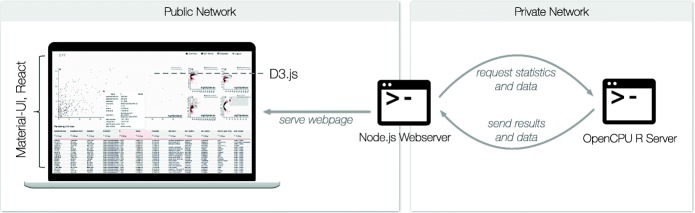
Implementation overview. A node server serves the front-end that is implemented in Javascript/HTML5 using the D3.js, Material-UI and React libraries. The node server provides each client with the data pulled from the OpenCPU back-end, which is only accessible for the node server. Heavy computational tasks are performed on the OpenCPU server. The node and OpenCPU server reside on one machine deployed using a *Docker* image. This architecture ensures that the potentially sensitive data is not accessible through the internet

The **front-end** was realized as HTML/CSS3/JavaScript (ES6) web-app (Fig. 1 left). Facebook’s React [9] library for JavaScript is the back-bone of the user interface and provides efficient means of building interactive components by distributing data changes throughout all user-interface components. The user interface components were built with Google’s Material Design [10].

D3.js [11] is employed for building the scatter- and hex plot visualizations and provides useful methods for handling transfer functions and axis labels.

The major challenge when implementing s ·nr was to minimize the number of rendered elements. Modern browser rendering engines, while already being heavily optimized, do not perform well given a high amount of displayed elements. Displaying 50.000 genes as SVG circle elements for 4 data sets in scatter plots renders any web-page unusable even on powerful machines. This calls for many optimization tweaks, such as binning scatter plot entries into hex plots and only rendering genes as circles after they are filtered. Another example is the data table, which behaves exactly like a classic HTML5 scrollable table, while it actually only renders the list elements of the current view pane with spacers above and below that are dynamically adjusted depending on the scroll position, reducing the number of rendered elements in the list from ∼50.000 to ∼20, depending on the resolution browser view frame size. This is facilitated with heavy use of React and D3.

The **back-end** is made of two components (Fig. 1).

**(1) Statistical computation**. The statistical computation back-end relies on the statistical programming language R. To provide functionality for importing, processing and serving data, we built the snR package for R. It also facilitates access to Ensembl Biomart through the biomaRt package [12] and handles the PCA calculation for the overview plot. Running the PCA calculation on a vast number of experiments is demanding and needs to be carried out as fast as possible to allow for an interactive workflow where users do not have to wait for minutes or hours to get the results. One major optimization we incorporate is saving the *public* experiments already in a matrix format that can be piped into the PCA algorithm. Only the *private* experiments have to converted on runtime, saving a couple of seconds. For further optimization, we keep the *public* experiment PCA input matrix in memory all the time removing the need of loading it every time when calculating a PCA, which, depending on the number of public experiments that are attached to s ·nr, can take up minutes. This, however, increases the footprint of the snR package in the system memory several gigabytes. In order to reduce the computation time of PCAs for large matrices from hours to seconds we employ a randomized single value decomposition algorithm provided by the RSVD package [13]. To avoid redownloading the required variables from the Biomart database, we cache them on disk as R dataset.

A second package, snR-GO, handles GO term analyses, which also relies on the cached data retrieved from the biomaRt package.

OpenCPU [14] exposes R methods using a RESTful API for method calls generating files including the JSON format which is easily readable by JavaScript. Unrestricted access to the RESTful API provided by OpenCPU would allow users to read all the data located on the system, yielding security issues when building a multi-user system. To solve this problem, we employ a second server described in the following paragraph.

**(2) Web-server and gatekeeper**. A custom-built Node.js server solves two tasks: (1) provide a web-server for the s ·nr web-app and (2) act as authenticator and gate keeper between the front-end and the R back-end. A major disadvantage when serving web-apps is that the app is accessible to everybody connected to the network where it is hosted. If the web-server is available to the world-wide-web, everybody can access the data through s ·nr. Therefore we implemented a user authentication system based on user tokens. The Node.js server stores user names, the files the user has access to as well as the bcrypt hashes [15] of the user’s password. On successful login, the user’s machine is provided with a token that has to be sent with every request on the server to check if it matches to the correct user. Users can access their own private experiments as well as all public repositories. A typical request looks as follows: A request for calculating a PCA is made from the client to the Node.js server through a POST together with the user’s token, which is then verified and if valid, the server will call the R command on the OpenCPU server. On completing the calculation, the Node.js server will retrieve the result as JSON file and pass it back to the client triggering the rendering of the PCA scatter plot.

We deploy s ·nr as **containerized solution**, which allows for easy sharing of data and methods as well as a well-defined and reproducible computing environment [16]. We provide a system to deploy s ·nr as a Docker container. The idea behind Docker is similar to virtual machines, it allows developers to create a system with installed software packages as well as data and provide it as an image. Using the Docker engine, users can convert the image into a container that is an exact replica of the exported system.

The Docker container both deploys (1) the Node.js and (2) OpenCPU server. The configuration is limited to the Docker image creation and customizing the Node.js server settings. Only the Node.js server needs to be accessible by the network, masking away all the data from public access and potential hacking attempts. Detailed instructions on how to setup s ·nr can be found in Additional file 5.

Having both the Node.js and OpenCPU server in one Docker container requires only a single machine to deploy s ·nr. Due to the demanding memory requirements of the snR package mentioned above this machine requires at least 15 GB of RAM. Support for multiple OpenCPU servers is not implemented since the most time-consuming operation is the PCA calculation which cannot be easily distributed among multiple servers and hence multiple R sessions.

## Results

In this section we describe the s ·nr interface and how we incorporate third-party services. A video tour of s.nr can be found in Additional file 6


Additional file 6: Video demonstration.mp4 — Video demonstration of s ·nr. A video showcasing the features of s ·nr. (MP4 100,558 kb)


Typically users want to examine one experiment in detail. We define this as the *focus experiment*. To be able to put it into context of other data, we extract and display experiments that carry valuable information when analyzing the *focus experiment*. We define these as *context experiments*.

### User interface design

The analysis focus is typically on one experiment. To put it into context of other data and observe patterns over multiple experiments, we include additional data. The user interface is divided into two major components. (1) The *overview visualization* shows which experiments express similar expression profiles and allows users to select experiments for further investigation. (2) The selected experiments can be analyzed in the *details view* that provides simple yet efficient means for displaying and querying the data as well as extracting GO terms. The selection of genes feeds back into the overview visualization, which can be triggered again to only consider the user-defined subset of genes and refines the search of functionally similar experiments.

**Fig. 2:**
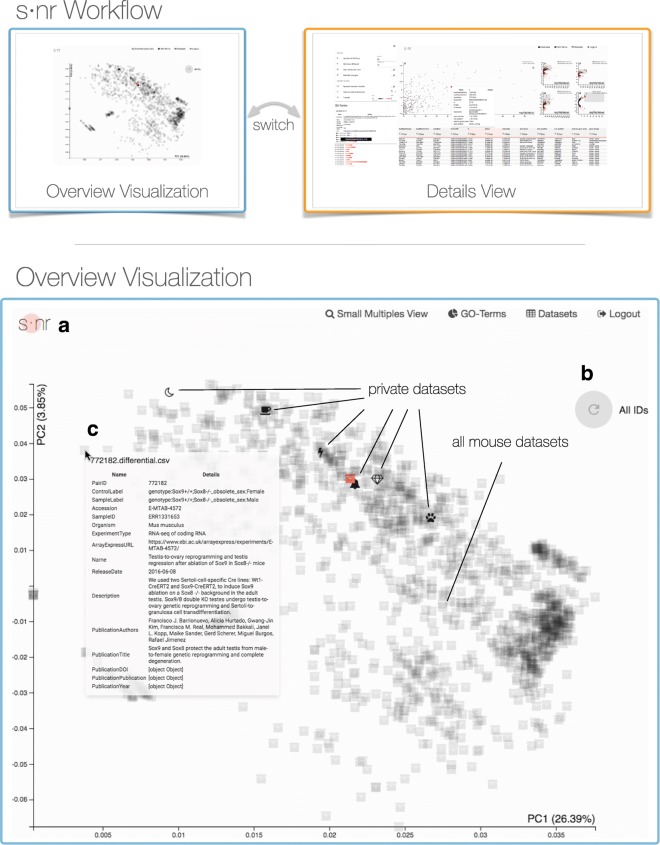
Workflow of s ·nr and detailed overview visualization showing a principal component analysis (PCA) of 1543 differential RNA-Seq expression mouse profiles. The s ·nr workflow consists of the overview visualization view that is used to select datasets based on similarity. The user can further investigate the selected data sets using the details view. Mean as an iterative analysis loop the user can always go back to the overview visualization to adjust the selection of potentially interesting data. We derived the data depicted in the overview visualization from ArrayExpress, processed it using QuickNGS, and provide the result with this paper. The analysis starts with the overview plot showing the first two PCAs of the *p*-values of all genes of public and private data sets. Public data sets are uniformly assigned the box icon and a higher transparency to facilitate identification of the user’s data. Data with similar *p*-values cluster together. **a** The dot of the s ·nr logo emits a fading circle when data is fetched from the server. **b** The PCA displayed is based on data for all genes. On brushing data sets in the details view, the user can narrow down the genes of interest and trigger a new PCA calculation based on the selected group of genes. **c** Mouse-over shows meta data of the data set. Clicking on a data set icon fetches its data and passes it to the details view. Icons of downloaded data are rendered orange, data sets flash upon loading

The starting point of a s ·nr session is the **overview visualization**. We want to provide the user with a birds-eye view on all the available data on starting s ·nr. We facilitate this by providing a scatter plot based on the first two Principal Components (PCs) calculated on the p-value of all genes from all experiments (Fig. 2). The view distinguishes *public* experiments derived from public repositories and *private* experiments, usually from the same lab or institute.

**Pictographic experiment representation**. Humans excel at distinguishing small simplified pictographic representations (icons). We utilize this by assigning an customizable icon to each experiment. This approach imposes two major advantages: (1) Avoid over-plotting due to labeling a vast number of datasets, and (2) the icon represents the experiments in the individual views and manifests a clear yet simple visual link. Due to the high count of *public* experiments when incorporating public data repositories they are all assigned a box icon and are rendered semi-opaque to highlight *private* experiments. The user can also customize the *public* experiment icons to create a cognitive link.

**Means of interaction**. A visual feedback of communication with the server is indicated with a emitted circle in the s ·nr logo (Fig. 2a). Calculating the PCA based on the currently selected genes is triggered with the only button integrated with the plot (Fig. 2b) Hovering over an experiment icon opens a context menu showing meta-data about the experiment (Fig. 2c). For *public* experiments, this also provides a link to the repository or the publication associated with the experiment to follow up more details. Clicking on a experiment icon pulls the associated data from the server and colorizes the icon indicating the loading status. The experiments can then be investigated further in the details view.

**Fig. 3:**
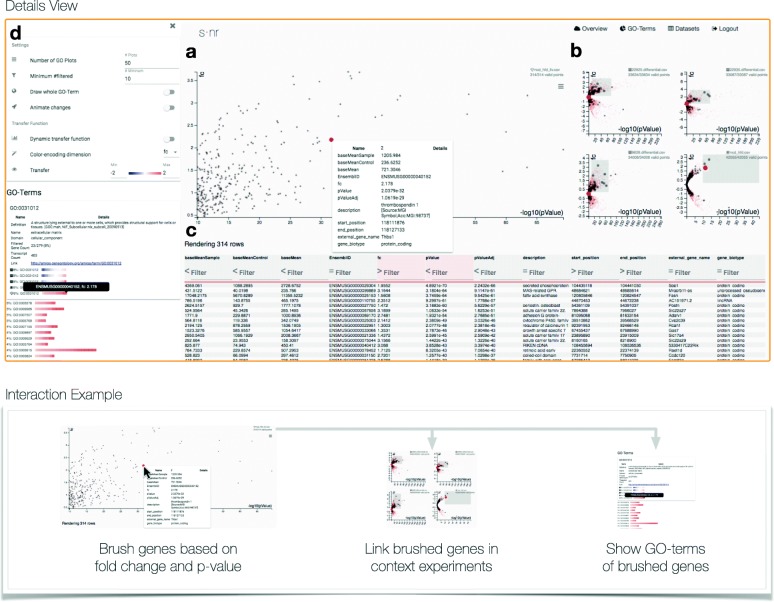
Details view for five mouse data sets with open GO term pane. The details view consists of three major interconnected components (**a**–**c**). Additionally, the GO term pane (**d**) is open. **a** The focus experiment is depicted in the large scatter/hex plot. **b** Further data sets are visualized in the small multiples of the large scatter plot. **c** The table view shows detailed information per gene for the main experiment. **d** The GO term pane shows GO terms for the selected genes and associated options. Opening a GO term displays additional information about the term as well as the expression of it in the *context* experiments. Each GO term is represented using a GO plot which can be customized in the panes options. We depict a typical interaction example at the bottom, where brushing (selecting) genes in the main scatter plot leads to highlighting the corresponding genes in the context experiments and also automatically triggers a GO-term analysis of the selection

The **details view** is the core of s ·nr. It consists of three components (Fig 3a–c).

**(1) Main scatter plot of the focus experiment**. The *focus experiment* is displayed as large scatter plot in the center of the user interface (Fig. 3a). Initially the scatter plot shows a volcano plot [17] showing −*log*_10_(*pValue*) against *foldchange*. To avoid a cluttered visualization when rendering all genes as dots we incorporate a binned aggregation visualization [18] dividing the space in hexes and counting the number of genes falling into each hex. The fill color of the hex maps represents the number of genes it contains. Genes are rendered as dots either when a gene is selected in a different view to show context information or when the total number of genes to render is below 5.000. The user has the option to always render genes as dots using the scatter plot option menu. The dots are rendered semi-opaque to counteract over-plotting, rendering populated areas darker. By spanning rectangles on click, the user can *brush* genes in the scatter plot. The default behavior is to zoom in on the selected genes to see the selected elements in detail and refine the selection further. The selection will be *linked* to all other views described below. On mouse-over a gene, that gene is *highlighted*, triggering a pop-up context window showing details about the gene, its statistics and meta-data. *Highlighted* genes are also *linked* to all views, showing as much context information as possible. Through various options, the user can change the transformation of the scale of the axes (linear, -linear, log2, -log2, log10, -log10, sqrt, -sqrt) as well as zooming behavior (always show whole range or only selected range) and rendering-related options such as always drawing genes as dots.

**(2) Small multiples of context experiments**. We want to display the *context experiments* in a way to show as much information related to the *focus experiment* as possible while occupying little screen and cognitive space (Fig. 3b). Small multiples [19] are a series of graphics sharing the same axis and scales allowing for easy comparison. We incorporate this idea for the *context experiments* to display them according to the *focus experiment* representation. The small multiples share the visual features of the main scatter plot rendering it a hybrid visualization of hex- and scatter plots. They also share the main plot x- and y-axis as well as the transformation. The icon in the heading of each small multiple allows to match it with the experiment it represents.

The strength of the small multiples approach becomes evident when a subset of genes is selected, e.g. by brushing them in the main scatter plot. The small multiples do *not* zoom into the selected area but rather show all selected genes as highlighted (more opaque and bigger) dots. This simple yet effective method allows users to rapidly check different sets of genes along multiple data sets. If, for example, only the up-regulated genes below a specific *p*-value in the default volcano-plot are selected, the small multiples show the regulation status in the *context experiments* (Fig 3a, b).

By clicking on the description header of a small multiple, that experiment will be set as *focus experiment* moving the current one to a *context experiment*. This allows for rapid switches between experiments when new hypotheses arise from the investigation. This is useful for investigating a potentially interesting public experiment that was found using the *PCA overview visualization* in detail.

**(3) Data table**. All interview partners reported extensive use of office spreadsheet applications for navigating and filtering experiments. While this may not be the most efficient way of looking at the data, it is still one the users of s ·nr potentially have years of experience in, rendering them efficient in consuming data through tabular representations. The data table view (Fig 3c) facilitates this interface and incorporates it into the whole analysis workflow as one essential steering point.

The data table view shows all available information for each entry of the *focus experiment*. Furthermore, filter can be applied through the input fields in the table header. The filter is made of two components, (1) the comparison operation button and (2) the input field. For numerical variables, the operation button can take the values <, > and =, which are applied on the input field. For example, input for p-value <0.005 selects all genes consisting of small p-values. Categorical variables only work on the = comparison, triggering a string matching on the input field. Analogously to filtering in the main scatter plot, the filter is applied to all visible representations, limiting the list to the filtered entries and highlighting the genes in the scatter plots.

Next to providing a tabular data representation and filtering on multiple dimensions at once, the data table view serves as input for the dimensions mapped to the x- and y-axis of the scatter plots. Clicking on a dimension name in the table puts it on the x-axis of the scatter plots and moves the current x-axis to the y-axis. This allows for rapidly changing the perspective on the experiments without adding user interface elements. Starting with the initial volcano plot, the user can for example change the plot to fold change against base mean with just two clicks. This works well with the adjustable scale transformation function of scatter plots. The table view can apply filter thresholds while the scatter plot also allows for defining filter ranges.

### Third-party service integration

The details view is supported by two panes addressing data access as well as interfacing third-party services.

**(1) Experiment selection pane**. The experiment selection acts as central hub for all *private* and *public* experiments. Selecting experiments downloads them from the server and puts them into the details view or removes them from it. The user can set the *focus experiment* and customize the experiment icons.

The Biomart selection allows to download additional information. Biomart [20] database provides rich information on transcript, gene, and protein level. The reason for s ·nr to require the EnsemblID dimension for each experiment is to enable Biomart annotations to be added. This allows us to keep the storage footprint on the back-end small by only saving information unique for the data set and attach all additional information on demand. The interface in the experiment selection pane allows the user to customize the information attached to the data set which can then be used to assess and filter the data further. Since the current implementation is restricted to gene-level information, we include Biomart variables only on gene level.

**(2) GO term pane**. GO term analysis was the most frequent request in the user study. When analyzing RNA-Seq data, users usually incorporate the web interfaces of services such as Panther [21]. This requires extracting the list of genes to filter into a specific format which is tedious and error-prone. The GO term pane (Fig. 3d) includes the GO extraction in the analysis workflow of s ·nr.

The GO terms are initially derived from the Biomart database. Upon gene subgroup definitions by filtering in any of the s ·nr views, the GO term pane will *automatically* retrieve the terms *containing the selected genes*. The resulting list of GO terms is sorted by percentage of selected genes in the term. This metric favors GO terms of small size (the number of genes associated with them). To mitigate this, the user can set a minimum and maximum size of GO terms considered for the calculation. The individual GO terms are represented using our *GO term plot*, where each gene is represented by a small rectangle that derives its color from a data dimension, usually fold change. The color scale ranges from blue (low limit) over white (mean) to red (high limit). The width of the plot is the sum of the width of the gene representation rectangles and is relative to the largest GO term fitting the gene selection query. When mapping fold change to the GO plot, the user gets an at-a-glance view of how many genes in the term are down- or up-regulated. Using the options pane, the user can adjust the dimension mapped to the GO plot as well as its transfer function. Additionally it allows for displaying all genes in the GO terms, not restricting it to the selected ones.

The GO plots are rendered in the overview of the pane for the *focus experiments*. On click the GO term expands with additional information such as name, definition, total gene- and transcript count, its entry on the Gene Ontology Consortium webpage as well as the GO plot for all *context experiments*. The experiment icons allow to associate the GO plot with the corresponding *context experiment*. Hovering the mouse over the GO plot highlights the gene under the cursor, showing its gene name as well as highlighting it in all other plots, triggering also the additional information context menu in the scatter plots.

## Discussion

As s ·nr is an endeavor of concurrently analyzing a large number of experiments, we discuss in this section how it performs computationally and as analytical tool. We then compare s ·nr to published related analysis tools.

### Performance

We have tested s ·nr with all publicly available RNA-Seq data for mouse mus musculus provided by the ArrayExpress platform [22]. The data was processed by the QuickNGS platform and consists of 2184 samples split up in 1543 pairwise experimental conditions. The data is supplied with the source code of this paper^1^. A demonstration instance of s ·nr can be accessed as well^2^.

The heavy computational processing is carried out using the OpenCPU/R server, which means s ·nr can only be installed on a capable machine. For the packages with public experiments provided with this paper, we recommend a computer with at least 15 GB of RAM. The PCA analysis takes about 20 s for this large amount of experiments and therefore displaying the initial *overview visualization* is sufficiently fast. Calculating the PCA on a subset of genes defined by the user significantly reduces the PCA computation time. Since both OpenCPU and node.js server are bundled in one Docker image, both of them run on the same machine. The client-side rendering scales well with the number of displayed data, the *details view* performance is dependent on the number of *context experiments*. We recommend a maximum of 4 *context experiments* even though the software performs well beyond that number.

### Application study

We conducted a Visual Data Analysis and Reasoning (VDAR) technique [23] to characterize the systems ability to generate and follow up hypotheses in the data. We carry out VDAR using a case study using the thinking-aloud technique to comprehend the reasoning and thought process of the user.

For our proof-of-concept evaluation we rely on **public data** for mus musculus provided by ArrayExpress [22]. The data was processed using the QuickNGS platform.

To test the practicability of s ·nr for VA data exploration and its suitability for deriving novel contextual insights by integrating private with public datasets in an unbiased manner, we (re)-analyzed two **private datasets** from an unpublished study on transcriptional regulation of hepatic gene expression under different states of nutrient abundances conducted in C57BL/6 mice. Here, we quantified DE genes in liver at the end of diet-induced obesity (DIO), which was elicited by chronic (24 weeks) of high-fat-diet (HFD) feeding, as compared to 24 weeks feeding with micronutrient-matched normal chow diets (NCD).

To put DIO DE gene changes into the context of normal liver physiology, we additonally quantified differentially expressed genes in liver after ad libitum feeding, 16 h fasting or 16 h fasting following by 6h refeeding, as the latter leads to well-understood expression changes in genes important for gluconeogenesis and lipid metabolism [24].

The exploration started with selecting the NCD/HFD liver data set from the lab as *focus experiment* as well as a few public data sets that were close to it on the overview visualization as *context*. Those data sets were then analyzed further in the small multiples view. By setting filters for fold change and p-value no pattern could be observed in the small multiples of the public *context experiments* (genes did not follow the same trend for up- or down regulation). Hence the *context experiments* were discarded. By switching back to the overview visualization and re-triggering the PCA to be only calculated on the differentially expressed genes. Based on the updated overview, three new data sets of interest clustered around the *focus experiment* were selected and assigned icons to:

1. E-ERAD-209 (a): Ad libitum versus dietary restriction, which is the opposite condition of the focus experiment
2. E-ERAD-209 (b): Ad libitum into dietary restriction vs dietary restriction in liver. This is the same dataset as above but regards to a different group comparison.
3. E-MTAB-3978: Mature adipocytes from white adipose tissue in conditions *preadipocyte*_*time*:1.0 *adipocyte*_*time*:30.0.

**Fig. 4:**
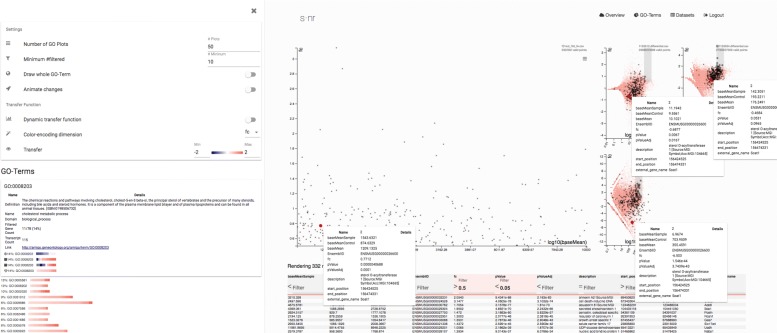
Data exploration showing baseMean against foldChange and expressed GO terms. Screenshot made during the application study of JWK. After selecting the displayed variables and transformation, JWK filtered all genes matching *pValue*<=0.05 and *foldChange*>0.05. JWK proceeded to look at the expressed GO terms, specifically the *cholesterol metabolic process* term. He highlighted the gene *Soat1* to show detailed information for that gene in all displayed data sets

These data were analyzed further in the details view, switching the standard volcano plot presentation to a visualization showing log10(*baseMean*) against *foldChange* by clicking the corresponding headers in the table (Fig. 4 right). This plot can be used to assess fold changes dependent on abundance. The user selected up-regulated highly abundant genes in the main scatter plot. Using the GO term pane the user assessed terms of the selection yielding terms such as cholesterol metabolic process, collagen trimmer, and steroid metabolic process. In the ad libitum compared to dietary restriction condition of the E-ERAD-209 (b) dataset, the user observed a inverse relationship in these terms using the GO term plot (Fig. 4 left). Up-regulated processed are down-regulated and vice-versa. While the experimental setup of E-ERAD-209 (b) is similar to the focus experiment, it is compared in the opposite direction, explaining the inverse relationship, showing that the observation makes biological sense and the similarity plot yields meaningful results. Analogously, the GO terms and differentially regulated genes of E-ERAD-209 (a) are regulated in the same direction as in the *focus experiment*.

### Related work

We compared s ·nr to five other tools that facilitate a visual exploration of RNA-Seq data. DEIVA, published in this journal [25], is closest to our approach by also providing a web-based platform that shows differential gene expression analysis based on a hexagonal binned volcano plot. DEIVA also allows to query data based on fold change, false discovery rate and baseMean using slider inputs, highlighting genes as well as assessing genes in a tabular representation. In order to assess the capabilities of s ·nr with regards to competing tools, we enhanced the comparison table incorporated by the DEIVA authors, see Table 1.

**Table 1:** Comparison of s ·nr to competing tools

Tool		s ·nr	DEIVA	DEGUST	Oasis	Glimma
Features	Locate & identify genes	✓	✓	✓	✓	✓
	Interactive plots	✓	✓	✓	✗	✓
	DE analysis pipeline	✗	✗	✓	✓	✗
	Databases	Biomart	✗	KEGG	✗	✗
	User data	✓	✓	✓	✓	✓
	Public data	✓	✗	✗	✗	✗
	Compare data	✓	✗	✗	✗	✗
	Show data similarity	✓	✗	✓	✗	✗
	Web-based	✓	✓	✓	✓	✗
	License	MIT	MIT	GPLv3	Closed source	LGPL
Dependencies	Development	node.js, R, Docker	node.js	Ruby	No custom deployment	R
	Server	15GB RAM with provided data			No custom deployment	

Major contributions of s ·nr are (1) the capability of analyzing multiple data sets and putting the data into context, (2) the feature to filter based on all variables, (3) the ability to display all numerical variables in the scatter plot and (4) calculation of similarity between differential gene expression datasets. Compared to the other tools, s ·nr has a significantly higher demand on the server’s RAM. This is because of the large size of data sets to be processed in a time-efficient way to facilitate real-time responses. Interactive analysis packages such as Glimma [26] allow for very basic filtering and selection of genes using text input, but lack the link between views and are require the user to load the data using R. This excludes many users of RNA-Seq technologies that do not have these skills. Other web platforms such as Oasis [27] aim at providing a web interface for the whole RNA-Seq pipeline including the alignment and differential analysis steps, but usually lack interactivity in the data exploration aspect. We focused solely on the exploration aspect to keep the tool lightweight in order to maintain a low cognitive workload on the user due to complexity of the user interface. Degust [28] also facilities differential expression analysis of input genes and also allows for comparisons of multiple conditions in a data set. It also allows for filtering using KEGG pathways, but lacks features to compare the results with other public or private data sets.

While the discussed tools differ a lot with respect to their feature set, all of them perform fast and responsive. Since comparing data and calculating similarity is unique for s ·nr, we cannot conduct a quantitative comparison of these features.

## Conclusion

We demonstrated that s ·nr provides interactive visual analysis methods for RNA-Seq data and puts them into the context with other private and public data. The client-server system allows to outsource computationally heavy operations on capable back-end machines which then transfer the result to the user’s front-end device. We showed that s ·nr performs well with high dataset counts by processing all publicly available mouse RNA-Seq datasets from the ArrayExpress platform which are also provided with this publication. The open input format of s ·nr allows users to pipe in results from any RNA-Seq based differential gene expression analysis algorithm. A containerized deployment allows for a simple setup procedure and allows s ·nr on any machine with a Docker instance. Researchers are able to extend s ·nr further with its permissive open source license.

## Availability and requirements

- **Project name**: s ·nr
- **Project home** https://github.com/snr-vis/
- **Operating system**(s): Platform independent
- **Programming language**: Javascript, R
- **Other requirements**: Docker
- **License**: MIT
- **Demo**: Login demo/demo: snr.sf.mpg.de (Tested with Google Chrome)
- **Any restrictions to use by non-academics**: none

## Additional files

Additional file 1 Interview Structure & Questionnaire.pdf — Interview structure and questionnaire. Detailed protocol on the questionnaire and structure guiding the interviews with the domain experts to assess requirements for s ·nr. (PDF 126 kb)

Additional file 2 Requirement Analysis Result.pdf — Requirement Analysis Result. Detailed description of requirement analysis for *user*, *task* and *context* for s ·nr. (PDF 179 kb)

Additional file 3 Data format.pdf — s ·nr data format. Detailed data format information used in s ·nr. (PDF 140 kb)

Additional file 4 Data dictionary.json — Example data dictionary. Example data dictionary specifying dimension names as well as meta data. (JSON 2 kb)

Additional file 5 Setup snr.pdf — Instructions for setting up s ·nr. Detailed instructions on how to setup a s ·nr instance. (PDF 259 kb)

## Abbreviations

DE: Differentially expressed
GO: Gene ontology
NGS: Next generation sequencing
PC: Principal component
PCA: Principal component analysis
RNA-Seq: Whole transcriptome shotgun sequencing

## Footnotes

1 Setup instructions and link to public files: setup-snr

2 Demo instance. Tested with Google Chrome (login demo/demo): snr.sf.mpg.de
