# Supplementary material for: s ·nr: a visual analytics framework for contextual analyses of private and public RNA-seq data
Source: BMC Genomics. 2019 Jan 24;20:85. doi: 10.1186/s12864-018-5396-0 (PMC6346532; doi:10.1186/s12864-018-5396-0)
Supplement: Supplementary file 2 — Interview Structure & Questionnaire.pdf — Interview structure and questionnaire. Detailed protocol on the questionnaire and structure guiding the interviews with the domain experts to assess requirements for s ·nr. (PDF 126 kb) [file 12864_2018_5396_MOESM1_ESM.pdf]

Interview Structure & Questionnaire

PAUL KLEMM, PETER FROMMOLT, JAN-WILHELM KORNFELD

2018-03-08

Contents

Interview Structure & Questionnaire **2**
Interview Structure 2
Questionnaire 3

Interview Structure & Questionnaire

Interview Structure

- *1. Introduction (5 Minutes)*
  - Goal: Explain the situtation and also state the scheduled time for the interview
  - Short introduction of ourselves as well as the goals:
    * We want to create a workflow for analyzing NGS data that provides integrated visualization and data mining techniques
    * The goal of the interview is to find out as much about your background, tasks and goals when analyzing NGS data and what the major challenges are
  - Ask if it is ok to record audio of the interview. It will not be played to anybody except the attendees of the interview!
- *2. Warm-Up (5 Minutes)*
  - Goal: Spend 5 minutes of small talk, make interview partner feed comfortable with the situation
  - Questions derived from the table below
- *3. Main Phase (45 Minutes)*
  - Goal: Derive as many information about user, task and context as possible
  - Questions derived from the table below
- *4. Cooling-off*
  - Goal: Make participant feel good about the interview and reduce cognitive load of all involved
  - Questions derived from the table below
- *5. Closure*
  - Thank interviewee for the time
  - Explain the further steps
    * We will compile a list of tasks and come up with ideas for our design
    * Prototypical implementations need to be tested
    * It would be helpful if interviewee can help here

Questionnaire

Question	User	Task	Context
Introduction (5 Minutes)			
Please explain briefly your scientific background and training	x	-	-
Where do you spend the majority of your work time? (computer, lab, somewhere else)	x	-	x
What is your main research question?	x	x	-
Warm-Up (5 Minutes)			
Main Phase (45 Minutes)			
NGS data: Who processes the data?	x	-	-
Which alternatives do you have when Peter does not analyze the data?	x	-	x
What format does the processed data have? (Excel)	-	-	x
Would you rather have it in a different format?	-	-	x
**Which major tools do you use for on the processed data?**	-	-	x
**Name three things that annoy you the most when analyzing or handling the data?**	-	x	-
**You have the tool to see precisely what you want in the data. What do you see?**	-	x	x
**What data analyses do you want to apply, but they take too much time**	-	x	-
**Which plots do you use to get insight into your data?**	-	x	x
Which plots do you use to communicate the results?	-	x	x
Cooling Off (10 Minutes)			
Do you discuss the data with anybody?	x	x	x
Which important finding did you get using NGS data analysis	x	x	x
Is it possible that I attend a analysis session to learn more about the approach?	-	-	-
Can we get a data set later on to include in our prototype?	-	-	-
