# Supplementary material for: s ·nr: a visual analytics framework for contextual analyses of private and public RNA-seq data
Source: BMC Genomics. 2019 Jan 24;20:85. doi: 10.1186/s12864-018-5396-0 (PMC6346532; doi:10.1186/s12864-018-5396-0)
Supplement: Supplementary file 3 — Requirement Analysis Result.pdf — Requirement Analysis Result. Detailed description of requirement analysis for user, task and context for s ·nr. (PDF 179 kb) [file 12864_2018_5396_MOESM2_ESM.pdf]

Requirement Analysis Result

PAUL KLEMM, PETER FROMMOLT, JAN-WILHELM KORNFELD

2018-03-08

Contents

Requirement Analysis Result **2**
User . . . 2
Uniform feedback . . . 2
Persona: Biologists without coding background . . . 2
Persona: Biologist with coding background . . . 3
Task . . . 3
Context . . . 4
Databases . . . 4
Tools . . . 4

Requirement Analysis Result

We conducted interviews with eight molecular biologists and derived Personas as well as information about *user*, *task* and *context* from the analysis.

User

Uniform feedback

- Getting trained in using new tools takes a lot of time
- `Excel` is the go-to tool for dealing with tabular data
- Hard cut-off for p-values and fold changes for significant regulations are set

Persona: Biologists without coding background

- Guidance required which tools and statistics can be incorporated
- Waiting time for results of computations very distracting
- File conversions between tools due to missing interfaces are tedious
- Relying strong on Bioinformatics output without checking the quality either because not empathetic to the problem or too tedious
- Quotes from the interviews:
  - *Analysis is very inefficient if you do not already have things in mind to search for*
  - *We do not look for surprises*
  - *Red flags should be raised actively-when weird things happen, the program should communicate this*

- Interested in integrated solutions containing all required bioinformatics tools in one place. These are for the most part commercial solutions, for example Geneious

Persona: Biologist with coding background

- File conversion not an issue (e.g. for piping names of differentially expressed genes into a web-service that expects a specific format)
- Use Galaxy to create genome analysis pipeline
- Do qPCR analysis and check whether genes cluster w.r.t. the phenotype
- Scientists go into programming to apply bioinformatics analysis methods themselves
- Achieve more freedom in exploring the data by incorporating `R` or `python`
- Knowledge of available packages, usually implemented in `R`

Task

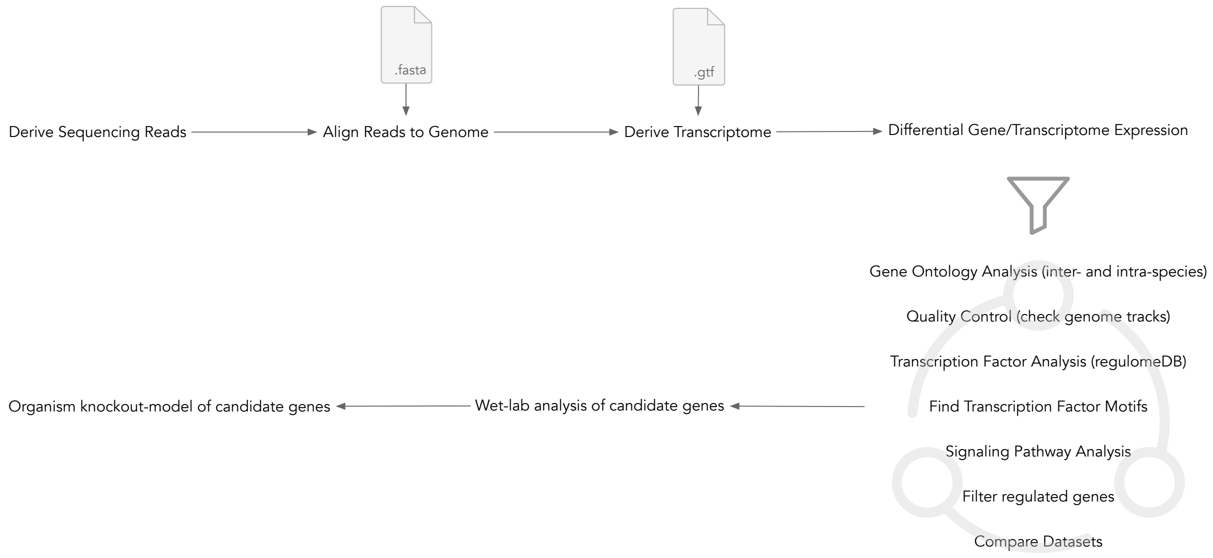

Figure 1: Analysis workflow for RNA-Seq data starting from deriving sequencing reads to the organism knockout-model of the candidate genes. We aim to tackle as many tasks in the analysis cycle (lower right) as possible.

An overview over the steps incorporated in the analysis of RNA-Seq files, please refer to Fig. 1.

- **!** Gene Ontology Analysis - Create gene homologs and search for human pathways - *Guidance for gene onthology analysis required* - it is hard to assess validity and significance of such methods
- Transcription Factor Analysis (regulomedb.org - see Tools)

- Transcription Factor Motifs (FIMO - Find Individual Motif Occurrences - see Tools)
- **!** Compare datasets for RNA-Seq and CHiP, though the latter is hard to achieve (CIRCOS) - Comparison for RNA-Seq is done by copying regulated genes for each data set into a overview table. Workflow is manual and error-prone
- **!** Quality control - Check Genome Tracks - Nobody looks on QC except something obvious is going wrong (e.g. marker gene indicating the phenotype is not expressed)
- Derive Signaling Pathways
- Filter for significant genes in spreadsheet app, search for genes that look familiar - Perfect result would be filtering the Top-10 list of most important genes and associated gene ontologies

Context

Databases

- Gene Expression Omnibus Browser (GEO) from NCBI
- Encode Project
- Sources for GO analyses are spread over multiple web-pages for different organisms. There is Flybase for fruit flies, Wormbase for C. elegans and others. This makes it hard to aggregate information in a standardized way for multiple species - Wormpath/Modpath - *"Wormpath is a tool to investigate molecular networks in the nematode Caenorhabditis elegans using information on genetic interactions provided by the Wormbase. It is organized as an interactive web service and the results can either be browsed online or downloaded to a local computer."* - **Specific for worms** - Panther - DAVID - TopGO
- ModENCODE - *"The modENCODE Project will try to identify all of the sequence-based functional elements in the Caenorhabditis elegans and Drosophila melanogaster genomes."*

Tools

- All: Excel
- Venny for creating Venn diagrams
- regulomedb.org - Use RegulomeDB to identify DNA features and regulatory elements in non-coding regions of the human genome by entering dbSNP IDs, Single nucleotides and chromosomal region. This is deprecated software though
- FIMO - Find Individual Motif Occurrences *"The program searches a database of sequences for occurrences of known motifs, treating each motif independently. Motifs must be in MEME Motif Format. The web version of FIMO also allows you to type in motifs in additional formats."*
- Galaxy
- GSEA Plots

- CIRCOS Plots for relationships between data set
- qPCR Analysis of gene expression
- Geneious, a commercial Bioinformatics hub providing de-novo assembly, variant calling, annotation, alignment trees and other tools. The interface looks like a rather awful Java app.
- SeqPlots is a R package: *"SeqPlots is a web browser tool for plotting average track signals (e.g. read coverage) and sequence motif densities over user specified genomic features. The data can be visualized in linear plots with error estimates or as series of heatmaps that can be sorted and clustered."*
