# Supplementary material for: s ·nr: a visual analytics framework for contextual analyses of private and public RNA-seq data
Source: BMC Genomics. 2019 Jan 24;20:85. doi: 10.1186/s12864-018-5396-0 (PMC6346532; doi:10.1186/s12864-018-5396-0)
Supplement: Supplementary file 4 — Data format.pdf — s ·nr data format. Detailed data format information used in s ·nr. (PDF 140 kb) [file 12864_2018_5396_MOESM3_ESM.pdf]

Data format

PAUL KLEMM, PETER FROMMOLT, JAN-WILHELM
KORNFELD

2018-03-08

Data format

We designed s·nr to derive insight into differential gene expressions of RNA-Seq data. This RNA-Seq centric approach represents a mere proof-of-principle approach using a widely obtained class NGS data. The bare minimum of such a table consists of the following attributes: `Gene Identifier`, `Mean Group A`, `Mean Group B`, `Fold Change` and `p-Value`. In s·nr we require each pairwise analysis (further on referred to as *experiment*) to be in one separate table saved as `Character Separated Value` (CSV) file. Of note, this analytical workflow can easily be modified to incorporate other NGS data types, e.g. from chromatin immunoprecipitation (ChiP) coupled to seq (ChiP-Seq) in the future. Data can provided for s·nr in open formats from DE quantification algorithms, such as DESeq2 [1]. Mandatory columns of each experiment comprise `p-value`, `fold change` and `Ensembl Stable Identifier` [2]. Users can specify the column names containing this information in a separate data dictionary, omitting the need to edit raw output of differential expression calculation algorithms. The data dictionary is also used to store metadata that will be displayed by s·nr for detailed information about the experiment (see Additional file 4). Standardizing the variable names is essential for comparing experiments. By relying on open data formats, s·nr is not restricted to any platform or differential expression calculation method as long as the above mentioned mandatory columns are present.

By processing all data, private or public, with the QuickNGS pipeline, we mitigate biases introduced by different read alignment and abundance estimation algorithms [3]. Due to the open format and data dictionary design, s·nr allows to compare results based on different bioinformatics pipelines, but we strongly advise against doing so.

1. Love MI, Huber W, Anders S. Moderated estimation of fold change and dispersion for rna-seq data with deseq2. Genome Biology. 2014;15. doi:10.1186/s13059-014-0550-8.

2. Aken BL, Ayling S, Barrell D, Clarke L, Curwen V, Fairley S, et al. The ensembl gene annotation system. Database. 2016;2016:baw093. doi:10.1093/database/baw093.

3. Robert C, Watson M. Errors in rna-seq quantification affect genes of relevance to human disease. Genome Biology. 2015;16. doi:10.1186/s13059-015-0734-x.
