# Supplementary material for: s ·nr: a visual analytics framework for contextual analyses of private and public RNA-seq data
Source: BMC Genomics. 2019 Jan 24;20:85. doi: 10.1186/s12864-018-5396-0 (PMC6346532; doi:10.1186/s12864-018-5396-0)
Supplement: Supplementary file 6 — Setup snr.pdf — Instructions for setting up s ·nr. Detailed instructions on how to setup a s ·nr instance. (PDF 259 kb) [file 12864_2018_5396_MOESM5_ESM.pdf]

Setup s·nr

PAUL KLEMM, PETER FROMMOLT, JAN-WILHELM KORNFELD

2018-03-08

Contents

Setup s·nr **2**
Setup . . . 2
Requirements . . . 2
Download Required Files . . . 3
Quickstart: Example Script . . . 4
Monitor Docker Container . . . 5
Download public data . . . 5
Development . . . 5
Run Node.js Server and Front-End in Developer Mode . . . 5
Launching Server Application with PM2 . . . 5
Show Server Logs . . . 6
Administration . . . 6
Generate User File . . . 6
Create new User Workflow . . . 6
Adding Data . . . 6
Additional Information . . . 7
Source Repositories of s·nr . . . 7

Setup s·nr

Always up-to date installation instructions are located in https://github.com/snr-vis/setup-snr

Setup

Requirements

- The only dependency for s·nr is docker. Please find the installation files for your system here: https://www.docker.com/community-edition.
- Make sure to have at least `10-15 GB` of memory available for docker
- The public files occupy `7 GB` of disk space
- We recommend using s·nr with Google Chrome

Download Required Files

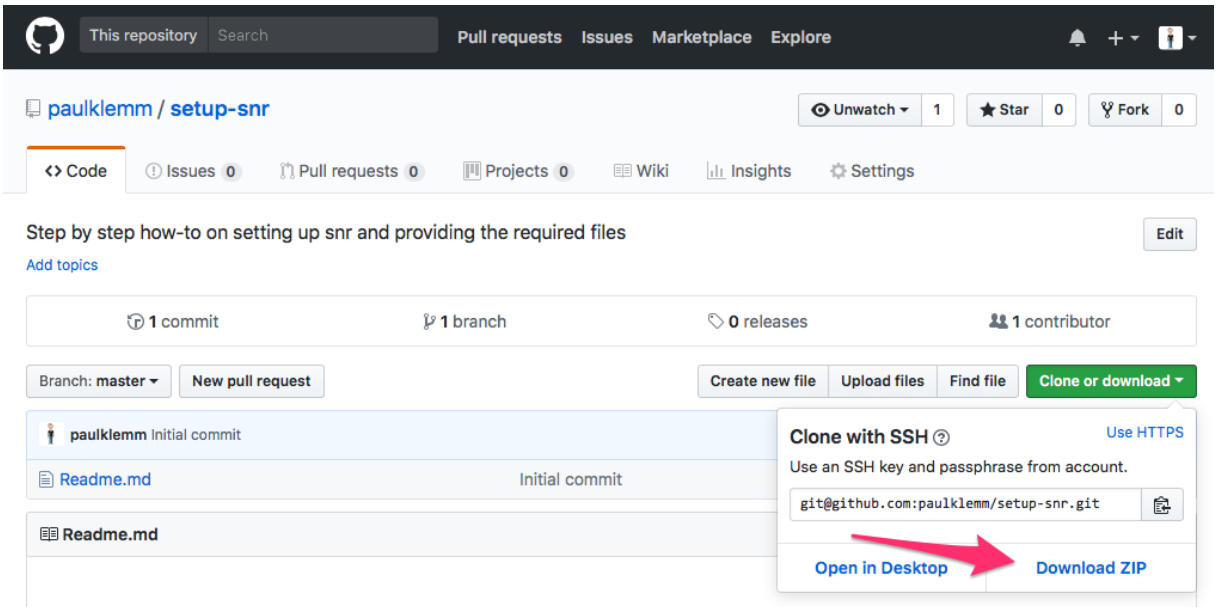

Figure 1: Download zip file of the project using GitHubs download function.

- Go to https://github.com/snr-vis/setup-snr and *either* clone the repository or download it as a zip (Fig. 1.).
- Go into the path of the downloaded repository and run the following commands:

```
# Download the public files
make
# Run docker
docker pull paulklemm/snr:paperrelease
docker run -t -d \
    -p 85:85 \
    -v $(pwd)/sonar:/home/opencpu/sonar \
    --name snr \
    paulklemm/snr:paperrelease
```

- Setting up the docker image can take a minute, depending on the system
- You can now access s·nr under **http://localhost:85/**
- Login: `user: demo | pw: demo`

Should you choose to expose the `OpenCPU` and `RStudio` instance running on the docker image, you can do so with the following call:

```
# Download the public files
```

```
make
# Run docker
docker pull paulklemm/snr:paperrelease
docker run -t -d \
    -p 85:85 \
    -p 8004:8004 \
    -v $(pwd)/sonar:/home/opencpu/sonar \
    --name snr \
    paulklemm/snr:paperrelease
```

This is not recommended and should only be used for development purposes because the R session of OpenCPU can access the whole docker file image and therefore all data stored there.

You find the servers at the following paths:

- <ip-of-docker-host-machine>:<port>/ocpu (e.g. http://localhost:8004/ocpu/)
- <ip-of-docker-host-machine>:<port>/rstudio (e.g. http://localhost:8004/rstudio/)
  - Login for RStudio User/PW: opencpu/opencpu

Quickstart: Example Script

This script requires a Unix (e.g. macOS or Linux) system with a running docker instance. It will download this repository and initialize s·nr.

```
# Setup snr directory
mkdir ~/snr
cd ~/snr
git clone https://github.com/snr-vis/setup-snr
cd setup-snr
# Download all public files
make
# Run the docker instance
docker pull paulklemm/snr:paperrelease
docker run -t -d \
    -p 85:85 \
    -v $(pwd)/sonar:/home/opencpu/sonar \
    --name snr \
    paulklemm/snr:paperrelease
```

Monitor Docker Container

We recommend `ctop` (https://ctop.sh/) for monitoring the activity and memory usage of docker container.

Download public data

The public data is available here https://edmond.mpdl.mpg.de/imeji/collection/RVILY1vK1naj8au9.

To add it to the bootstrap file system of this repository run `make` which will download the files to `<repo-path>/sonar/data/quickngs`.

Development

Run Node.js Server and Front-End in Developer Mode

For development it prooves easier to run a instance of the `node.js` server and the react front-end app outside of the `docker` container.

For this you are required to run a docker instance exposing the `OpenCPU/RStudio` port using:

```
docker run -t -d \
    -p 8004:8004 \
    -v $(pwd)/sonar:/home/opencpu/sonar \
    --name snr \
    paulklemm/snr:paperrelease
```

Now, clone the `node.js` and front-end repo https://github.com/snr-vis/snr and edit the `server_settings.json` to point to the appropriate OpenCPU path (edit `"opencpuPath": "http://localhost:8004"` line).

If you run the docker image on a separate machine that is behind a firewall you might forward the port using a command like `ssh -L 8004:localhost:8004 pklemm@aligner cat` to map the port to your `localhost`.

Launching Server Application with PM2

The docker container launches the `node.js` server as well as the `react` front-end using the `PM2` process manager. `PM2` can also be used for developement, whereas `pm2 monit` and `pm2 status` are most valuable tools to track console outputs and error messages of the processes. See details in the dockerfile on how to start `pm2`.

Show Server Logs

Error messages are printed out by PM2. To access them, please attach a pm2 monit session to the already running docker container using the following command (assuming the name of the docker container is “snr”):

```
docker exec -it snr pm2 monit
```

To see if pm2 is running properly, run:

```
docker exec -it snr pm2 status
```

Administration

Generate User File

The server’s API allows the create a user file by specifying path and password by calling: http://<url_to_server>:<port>/api/makeuserfilejson?pw=mypassword&path=/home/opencpu/sonar/data. You can use the response to create or edit the user files.

Create new User Workflow

1. Create a new folder for the user in the folder that is linked to the docker R back-end and add the data there
2. Create a dictionary.json file in that folder (see Structure of Data and OpenCPU Sessions)
3. Check server_settings.json file where the user configuration files are
4. Go to this directory and save the output of http://<url_to_server>:<port>/api/makeuserfilejson?pw=<user_password>&path=<path_to_data_on_r_back_end> to <username>.json
5. Log in to sonar with the new account

Adding Data

Create a folder for the user that matches the path in the user configuration file and add the data here. Note that the folder should contain a dictionary.json file for fallback settings for all files within this folder. You can also add custom dictionary files per dataset by creating a .json dictionary with the same name as the added file. See the demo user folder for an example.

Additional Information

Please refer to the readme of the `snr` repository (https://github.com/snr-vis/snr) for more details.

Source Repositories of s·nr

Home of the repositories is https://github.com/snr-vis.

The source code of s·nr is distributed on these repositories:

Link	Description
https://github.com/snr-vis/snr	`Node.js` back-end server and `React` front-end
https://github.com/snr-vis/snr-docker	`Dockerfile` for creating the docker image
https://github.com/snr-vis/snrgo	GO-term analysis `R` back-end package
https://://github.molgen.mpg.de/paulklemm/snR	`R` back-end package. *Repo on enterprise github because GIT Large File Storage support requirement*
https://github.com/snr-vis/setup-snr	Setup instructions for running a s·nr instance
